# Dynamics of Long-Range Temporal Correlations in Broadband EEG During Different Motor Execution and Imagery Tasks

**DOI:** 10.3389/fnins.2021.660032

**Published:** 2021-05-28

**Authors:** Maitreyee Wairagkar, Yoshikatsu Hayashi, Slawomir J. Nasuto

**Affiliations:** ^1^Brain Embodiment Laboratory, Biomedical Engineering, School of Biological Sciences, University of Reading, Reading, United Kingdom; ^2^Biomechatronics Laboratory, Department of Mechanical Engineering, Imperial College London, London, United Kingdom; ^3^Care Research and Technology Centre, The UK Dementia Research Institute (UK DRI), London, United Kingdom

**Keywords:** movement execution, motor imagery, electroencephalography (EEG), long-range temporal correlation (LRTC), broadband EEG, brain-computer interface (BCI), movement classification

## Abstract

Brain activity is composed of oscillatory and broadband arrhythmic components; however, there is more focus on oscillatory sensorimotor rhythms to study movement, but temporal dynamics of broadband arrhythmic electroencephalography (EEG) remain unexplored. We have previously demonstrated that broadband arrhythmic EEG contains both short- and long-range temporal correlations that change significantly during movement. In this study, we build upon our previous work to gain a deeper understanding of these changes in the long-range temporal correlation (LRTC) in broadband EEG and contrast them with the well-known LRTC in alpha oscillation amplitude typically found in the literature. We investigate and validate changes in LRTCs during five different types of movements and motor imagery tasks using two independent EEG datasets recorded with two different paradigms—our finger tapping dataset with single self-initiated asynchronous finger taps and publicly available EEG dataset containing cued continuous movement and motor imagery of fists and feet. We quantified instantaneous changes in broadband LRTCs by detrended fluctuation analysis on single trial 2 s EEG sliding windows. The broadband LRTC increased significantly (*p* < 0.05) during all motor tasks as compared to the resting state. In contrast, the alpha oscillation LRTC, which had to be computed on longer stitched EEG segments, decreased significantly (*p* < 0.05) consistently with the literature. This suggests the complementarity of underlying fast and slow neuronal scale-free dynamics during movement and motor imagery. The single trial broadband LRTC gave high average binary classification accuracy in the range of 70.54±10.03% to 76.07±6.40% for all motor execution and imagery tasks and hence can be used in brain–computer interface (BCI). Thus, we demonstrate generalizability, robustness, and reproducibility of novel motor neural correlate, the single trial broadband LRTC, during different motor execution and imagery tasks in single asynchronous and cued continuous motor-BCI paradigms and its contrasting behavior with LRTC in alpha oscillation amplitude.

## 1. Introduction

The brain activity is composed of various complex processes that undergo changes during different tasks and brain functions. Spontaneous electroencephalography (EEG) contains rhythmic oscillatory components such as delta, theta, alpha, and beta oscillations in narrow frequency bands, and arrhythmic scale-free broadband component without a characteristic timescale or frequency that leads to a typical 1/*f* EEG spectrum (He, 2014). Additionally, EEG also contains event-related potentials that are one-off non-oscillatory and non-rhythmic responses to sensory, cognitive, or motor events. During voluntary movement, distinct changes occur in all the above three components of EEG. In the rhythmic sensorimotor oscillatory component of EEG, we observe the well-known event-related (de)synchronization (ERD/S), which quantifies increase or decrease, respectively, in band power of narrowband sensorimotor oscillations during a motor task with reference to its baseline (Pfurtscheller and Lopes Da Silva, 1999; Yuan and He, 2014; He et al., 2015). In the event-related potential component of EEG in response to a motor task, we observe non-oscillatory non-rhythmic movement-related cortical potentials (MRCP), which are characterized by an increase in slow negative potentials (Shibasaki and Hallett, 2006; Bai et al., 2011). Changes in the arrhythmic broadband component of EEG during motor task, however, are not investigated in the literature.

Rhythmic narrowband oscillatory processes and arrhythmic broadband processes co-exist in EEG, where rhythmic oscillations appear as distinct peaks (e.g., alpha peak around 10 Hz) on the arrhythmic broadband 1/*f* EEG spectrum (illustrated in Wairagkar et al., 2019). However, there is more focus on studying rhythmic oscillatory components of EEG. The broadband arrhythmic process was previously considered as background noise in brain activity. However, recent reports suggest that the broadband activity has physiological and functional relevance (He, 2014), its dynamics change with task demand and cognitive state, and it has also been associated with the excitation/inhibition balance of the neuronal populations (Chaudhary et al., 2017; Haller et al., 2018). This has rekindled the interest to investigate the broadband EEG during motor tasks. Widely used complementary neural correlates of ERD/S and MRCP do not describe the dynamical changes in the temporal dependencies in the broadband arrhythmic EEG. Hence, we propose that changes in the broadband scale-free arrhythmic component in EEG can reveal yet another complementary neural correlate of voluntary movement (Wairagkar et al., 2019).

We previously studied the temporal dynamics of broadband EEG and found that its autocorrelation decayed slower during movement intention and execution than in the resting state (Wairagkar et al., 2018). We modeled these broadband autocorrelation dynamics on single trial EEG using the autoregressive fractionally integrated moving average (ARFIMA) model, which led to the discovery that broadband EEG contains coexisting short-range and long-range temporal correlations. These short-range and long-range temporal correlations changed significantly during voluntary movement and can be used together as novel neural correlates of movement (Wairagkar et al., 2019). Several studies have approximated short-range correlations using autoregressive models to estimate movement correlates from EEG (Schlögl et al., 2005; D'Croz-Baron et al., 2012; Wang et al., 2018). However, there is limited literature on long-range temporal correlations (LRTC) in broadband EEG (Hou et al., 2017; Lombardi et al., 2020), and there are no other studies investigating long-range temporal dynamics of single trial broadband EEG during motor tasks. Hence, in this study, building upon our previous work, we delve deeper to investigate changes in broadband LRTC during movement and compare it with the well-known alpha oscillation LRTC.

Neural activity has been reported to produce long-range interactions leading to power-law scaling, suggesting that these neuronal processes are similar across different scales (Kello et al., 2010; Berthouze and Farmer, 2012; Heiney et al., 2021). The power-law scaling is observed in several cases of neuronal recordings such as neuronal firings (Hu et al., 2013), neuronal avalanches (Benayoun et al., 2010; Palva et al., 2013), intracranial recordings such as local field potentials (Benayoun et al., 2010), and electrocorticography (Chaudhary et al., 2017), and non-invasive scalp recordings of EEG and magnetoencephalography (Nikulin and Brismar, 2005; Benayoun et al., 2010; Kwok et al., 2019; Jannesari et al., 2020) in both oscillatory and non-oscillatory processes. In the surface-level brain activity such as EEG and magnetoencephalography, the power-law scaling is observed in the form of the 1/*f* power spectrum of non-oscillatory or arrhythmic scale-free neuronal activity. Spontaneous oscillatory neuronal processes also show LRTC in their amplitude envelope fluctuations (Linkenkaer-Hansen et al., 2001; Nikulin and Brismar, 2005; Berthouze et al., 2010; Hardstone et al., 2012). LRTCs are the result of power-law decay of the autocorrelation of neural activity. The LRTCs have been observed in the alpha, beta, theta oscillation amplitude envelopes (Berthouze et al., 2010), alpha oscillation phase (Botcharova et al., 2014), broadband phase synchrony (Kitzbichler et al., 2009), avalanches (Benayoun et al., 2010; Palva et al., 2013), and energy profile (Parish et al., 2004; Benayoun et al., 2010). It is commonly postulated in the literature that the power-law behavior and LRTCs occur because the brain operates at criticality (Poil et al., 2012; Massobrio et al., 2015), thus optimizing information storage capacity (Kitzbichler et al., 2009) and enabling quick adaptation to the cognitive processing demands (Ezaki et al., 2020; Ouyang et al., 2020; Zimmern, 2020). In the absence of long-range temporal correlations, correlations on shorter timescales lead to reduction in the ability to integrate information (for example, during certain stages of sleep; Meisel et al., 2017).

In EEG, LRTCs have been traditionally identified in the amplitude envelope fluctuations of narrow frequency bands corresponding to different brain oscillations (Linkenkaer-Hansen et al., 2001; Nikulin and Brismar, 2005). LRTCs have also been observed in the sensorimotor oscillations (Linkenkaer-Hansen et al., 2004; Botcharova et al., 2015). LRTCs in the alpha amplitude envelope fluctuations decrease due to the disruption caused in the long-memory process by an external stimulus (Linkenkaer-Hansen et al., 2004; Botcharova et al., 2014; Zhigalov et al., 2016). Neurological conditions also affect LRTCs (Parish et al., 2004; Ros et al., 2014, 2016). The LRTCs can be modulated using neurofeedback where these correlations increase because of the closed-loop stimulus (Ros et al., 2014, 2016; Zhigalov et al., 2016). The scale-free dynamics are also identified in behavioral data (Palva et al., 2013). LRTCs in neuronal activity and movement patterns are correlated (Hu et al., 2004, 2013), and neural scale-free dynamics can predict the performance of motor tasks (Samek et al., 2016). There are very few studies in the literature that consider broadband LRTC such as the ones by Hou et al. (2017) that found attenuation in broadband LRTC during depression and by Lombardi et al. (2020) that characterized LRTC in the resting-state broadband EEG using neuronal avalanches. However, there have been no previous external studies investigating LRTCs in broadband arrhythmic EEG during different motor tasks and their relationship with alpha oscillatory LRTC, which is the focus of this study.

LRTCs are typically characterized in the amplitude envelope fluctuations of oscillations in brain activity. These oscillations are typically extracted using Fourier-based spectral methods. Cole and Voytek (2018) showed that the brain rhythms are non-stationary and not strictly restricted to pre-selected narrow sinusoidal frequency bands; therefore, restricting to a narrowband analysis can disregard important features present in the entire power spectrum. Hence, the LRTCs computed from narrow frequency band amplitude envelope fluctuations may not give complete information present in these brain rhythms, and there is a need for assessing LRTCs in the broadband arrhythmic brain activity as well.

The LRTC in EEG can be characterized by its scaling exponent computed from the spectral domain by estimating the slope of 1/*f* power spectrum on a log-log scale and computed from the temporal domain by fitting the power-law directly to the autocorrelation, both of which are often difficult to achieve in practice (Rangarajan and Ding, 2000; Delignieres et al., 2006). Hence, LRTCs are most preferably characterized in the temporal domain using Hurst exponent, which shows a consistent relationship with scaling exponents from autocorrelation and 1/*f* spectrum for a stationary time series (Rangarajan and Ding, 2000). A Hurst exponent between 0.5 and 1 indicates the presence of LRTC (Hardstone et al., 2012). The detrended fluctuation analysis (DFA) (Peng et al., 1995) is the most common method for estimating Hurst exponent in a non-stationary signal, which is computed from the slope of fluctuations in the signal at different timescales. The power spectrum analysis is not suitable for reliably identifying LRTC in a non-stationary time series (Linkenkaer-Hansen et al., 2001). The DFA is used for estimating Hurst exponent from EEG because it facilitates the detection of LRTC embedded in a non-stationary time series by avoiding artifactual dependencies caused by non-stationarity and trends (Peng et al., 1995; Kantelhardt et al., 2001; Linkenkaer-Hansen et al., 2001; Delignieres et al., 2006; Hardstone et al., 2012).

LRTC is considered an invariant property of brain dynamics spanning several time scales (Delignieres et al., 2006) and hence is not computed as a function of time. LRTC is traditionally estimated on an amplitude envelope of narrowband EEG oscillations, which requires long EEG segments (Linkenkaer-Hansen et al., 2001). With this approach, we cannot observe the ongoing instantaneous changes in LRTC. Detecting short movement from LRTC requires evaluating the changes in the dynamics of the LRTC continuously as a function of time. Berthouze and Farmer (2012) have previously captured the changes in LRTCs using a Kalman filter, but the timescales over which LRTCs were observed were still several seconds long. Here, we investigate the instantaneous changes occurring in the LRTCs using shorter timescales to study the fast brain dynamics during different motor tasks such that it can be applied in brain–computer interface (BCI). Continuous characterization of LRTC on short broadband EEG windows using BCI-style processing pipeline can enable detecting movement on a single-trial basis without the need of choosing participant-specific parameters. Our previous work (Wairagkar et al., 2019) has established the presence of LRTC during finger tap voluntary movement. Consistently, we obtained high classification accuracies to detect finger tap intention using broadband LRTC-related indices. In this study, we expand this investigation to explore the changes in the broadband LRTC in different types of movement and motor imagery with paradigms that are commonly used in BCI. To our knowledge, LRTCs in the broadband have not been observed before during motor imagery. Our analysis will help in understanding the functional role the broadband arrhythmic brain activity plays in motor command generation and motor imagery.

The aims of this paper are (1) to build upon our previous work to investigate further the dynamics of LRTC in single trial broadband EEG during five different types of motor tasks with different experimental paradigms including single asynchronous finger tap, and continuous fist and feet movement and motor imagery from two independent EEG datasets, validate broadband LRTC rigorously and demonstrate its generalizability, robustness, and reproducibility; (2) to compare and contrast the broadband LRTC with LRTC in the alpha oscillation amplitude envelope commonly observed in the literature during corresponding motor tasks to identify contrast in the dynamics of coexisting oscillatory and arrhythmic scale-free processes; (3) to classify movement intention, execution, and motor imagery from resting state by using broadband LRTC independently on a single-trial basis as a novel feature for applications in BCI.

## 2. Methods

### 2.1. EEG Datasets and Participants

The first dataset is our voluntary single finger tap EEG dataset (available from Wairagkar, 2017) that we recorded from 14 healthy participants (8 female, age 26±4 years, 12 right-handed). Ethical approval for the EEG experiment was obtained from the ethics committee of the University of Reading, UK. Informed written consent was obtained from all participants.

The second dataset is a publicly available EEG Motor Movement/Imagery Dataset (EEGMMI) (Schalk et al., 2004) from PhysioNet (Goldberger et al., 2000). This dataset is used to validate our novel broadband LRTC neural correlate and to widen its scope by extrapolating its utility for broader use in different types of motor tasks. EEGMMI dataset comprises EEG recorded from 109 participants for four different types of motor tasks: (1) right and left fist continuous opening and closing, (2) motor imagery of right and left fist continuous opening and closing, (3) both feet continuous movement and both fists continuous opening and closing, and (4) motor imagery of both feet continuous movement and both fists continuous opening and closing.

Our EEG dataset has self-initiated asynchronous single finger tap in contrast to the EEGMMI dataset, which has cued continuous fist and feet movement and imagery. Thus, these datasets cover complementary paradigms and motor tasks to assess the robustness and reproducibility of our broadband LRTC neural correlate for broader applications.

### 2.2. Experimental Paradigm and Pre-processing

**Figure 1:**
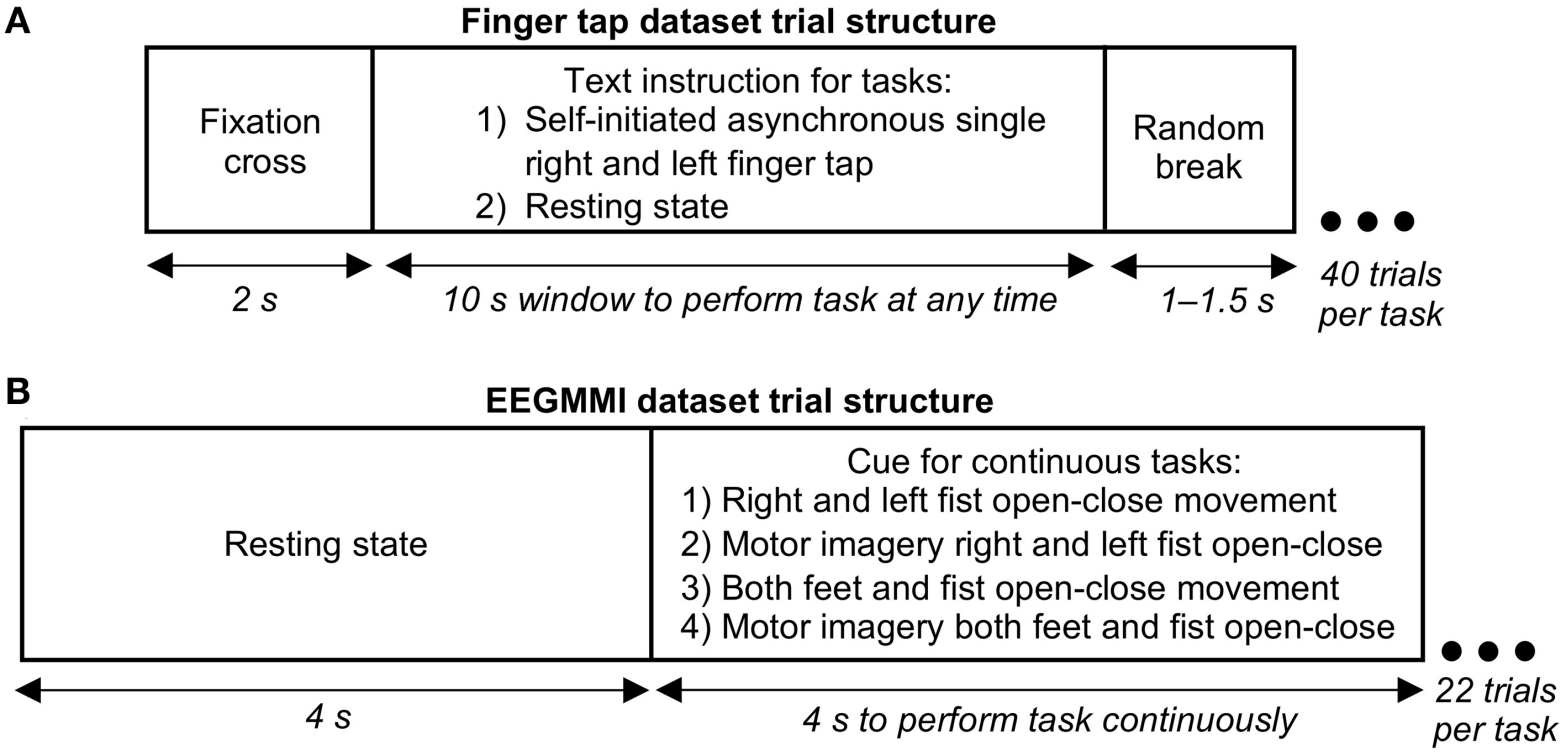
Structure of a single electroencephalography (EEG) trial in our finger tapping dataset and EEG Motor Movement/Imagery Dataset (EEGMMI) dataset. **(A)** Trial structure for single self-initiated asynchronous finger tap. Participants were given a window of 10 s to perform a single tap any time, or if it was a resting state task, they were asked to remain still with eyes open without thinking of movement. **(B)** Trial structure for four cued EEGMMI tasks, including continuous movement or motor imagery for 4 s of single or both fists and both feet.

Our finger tap EEG dataset was recorded for a self-initiated single asynchronous index finger tapping task. A text instruction was shown on the screen placed 1 m from the participant to perform a right finger tap, left finger tap, or resting state (no tap) within a following 10 s window asynchronously at any random time (see Figure 1A). Participants were specifically asked not to react immediately to the instruction to avoid cue effect. The timing of the initiation of the movement was entirely participant's decision. Forty trials per condition were recorded with the sampling frequency of 1,024 Hz and were downsampled to 128 Hz. The exact onset of finger tap was recorded using a microcontroller device and was co-registered with the EEG. Further details of the finger tap dataset are given in Wairagkar (2017).

The EEGMMI dataset recorded four cued motor tasks described in the previous section, including continuous fist and feet movement execution and motor imagery. Each trial started with a resting state of 4 s followed by a cue to perform continuous movement/imagery for 4 s (left/right arrows for respective fists, up arrow for both fists together, and down arrow for both feet together) as shown in Figure 1B. A 1 min baseline EEG with eyes open is included in the dataset, which we segmented and used as neutral state trials for further processing. There were around 22 trials for each of the four motor tasks per participant recorded with the sampling frequency of 160 Hz, which were downsampled to 128 Hz.

The same artifacts removal and pre-processing pipeline was used for both datasets. Artifacts were removed with independent component analysis (Jung et al., 2000) using EEGLAB toolbox (Makeig et al., 1997; Delorme and Makeig, 2004) before trial segmentation. EEG was bandpass filtered between 0.5 and 45 Hz using a fourth-order zero-phase non-causal Butterworth filter to avoid phase distortions. We extracted 6 s EEG trials (−3 to +3 s of movement onset) from our finger tap dataset and 7 s EEG trials (−3 to +4 s of motor cue) from the EEGMMI dataset from the channels C3, Cz, and C4 over motor cortex according to the 10–20 international system. These channels were selected for the study because of their locations on sensorimotor area responsible for hand and feet movement and imagery (Resniak et al., 2020). These trials were divided into 2 s sliding Hanning windows shifted by 100 ms. Each feature at time *t* was causally obtained on a window from *t* − 2 s to *t* s. All the analysis was done offline in MATLAB (The MathWorks, Inc., Natick, MA, USA).

### 2.3. Detrended Fluctuation Analysis to Identify LRTC in EEG

We hypothesized that the LRTCs in the broadband EEG change during movement intention and execution. We quantified the broadband LRTC using Hurst exponent computed using DFA (Peng et al., 1995). The DFA analysis calculates root mean square (RMS) fluctuations of integrated and detrended time series at different timescales as follows:

1. The time series *x* of length *N* is integrated according to Equation (1) where *k* = 1, ..., *N* and *y* is the integrated time series.
  (1) $$\begin{array}{r} y\left(k\right)=\overset{k}{\underset{i=1}{\sum }}x\left(i\right)-\bar{x} \end{array}$$
2. The integrated time series *y* is then divided into *N*/*n* non-overlapping boxes of length *n*, where *n* is individual timescales at which we want to compute fluctuations. The box sizes have an impact on the DFA scaling exponent and are usually chosen between *n* = [10, *N*/4] (Delignieres et al., 2006; Botcharova et al., 2013) to get good estimate of RMS fluctuations at each scale with [*N*/10, 4] number of boxes. We used 25 box sizes between *n* = [10, *N*/4] equidistant on *log*_2_ scale as our number of samples was a power of 2.
3. At each scale *n*, for every non-overlapping segment of *y* of length *n*, trend is obtained by least square linear fit. The *y*_*n*_ is concatenation of trends at a scale *n* for all the *N*/*n* boxes and the RMS fluctuations are computed according to Equation (2).
  (2) $$F(n)=\sqrt{\frac{1}{N}\sum_{i=1}^{N}(y(i)-y_{n}(i){)}^{2}}$$
4. A log–log plot of fluctuations at each timescale *n* (*log*_2_*F*(*n*) vs. *log*_2_*n*) was plotted and DFA scaling exponent was obtained by calculating the slope of the linear fit to this plot.

Since *N* is not divisible by *n* for each box size, fluctuations were obtained from performing the above analysis from forward and backward direction (Kantelhardt et al., 2001) of each EEG window and then averaging them at each timescale. When the log–log DFA plot is linear, the DFA scaling exponent or Hurst exponent indicates power-law in fluctuations at different timescales.

#### 2.3.1. LRTC Using DFA in Broadband EEG and Alpha Oscillation Amplitude Fluctuations During Different Motor Tasks

We studied the changes in LRTC in the broadband EEG, and compared and contrasted them with the LRTC in the alpha oscillation amplitude envelope during movement, which is typically assessed in the literature (Linkenkaer-Hansen et al., 2004). We investigated changes in the broadband LRTC and alpha envelope LRTC during different types of motor execution and imagery from the finger tap and EEGMMI datasets using the same analysis pipeline as described in the next sections. For clarity, throughout the paper, we use *H*_*BB*_ to indicate DFA scaling exponent or Hurst exponent in the broadband and *H*_*alpha*_ to indicate Hurst exponent in the amplitude envelope of the alpha oscillations.

### 2.4. Broadband LRTC in Single Trials During Different Motor Tasks

We performed DFA on each 2 s sliding broadband (0.5–45 Hz) EEG window shifted by 100 ms to obtain continuous changes in the LRTC (Hurst exponent *H*_*BB*_) throughout the trial during different motor tasks. The Hanning window was applied to each 2 s window to avoid edge effects. Note that 256 samples were available for performing DFA on a 2 s window. Delignieres et al. (2006) have shown that the DFA method can accurately estimate Hurst exponent in short time series. Our range of timescales ([10, *N*/4] samples, i.e., [78 ms 0.5 s]) is within the range suggested by Li et al. (2018) [*max*(*k* + 2, *F*_*s*_/*F*_*max*_), *min*(*N*/4, *F*_*s*_/*F*_*min*_)] where *k* = 1 (linear detrending in DFA) for filtered data between *F*_*min*_(0.5 Hz) and *F*_*max*_ (45 Hz). We used an exponential smoothing filter to smooth the *H*_*BB*_ in consecutive windows in single trials to avoid noisy estimates of *H*_*BB*_. We rigorously validated the scaling exponents *H*_*BB*_ and the presence of LRTC in broadband using the procedure described below.

#### 2.4.1. Validation of Broadband LRTC

We validated our results to confirm that the obtained broadband LRTCs were not artifactual. The autocorrelation of a time series decays exponentially if it has short-range dependence and slower than exponential if it has long-range dependence (Botcharova, 2014). A specific case of long-range dependence is LRTC, where the autocorrelation decays according to the power-law that is identified using the Hurst exponent. Hence, to identify LRTC, we must validate the Hurst exponent obtained using DFA. We systematically validated the LRTC in three stages as follows.

##### 2.4.1.1. Identification of Significant Correlations in Broadband EEG Using Surrogate Test

We first identified whether significant temporal correlations are present in the broadband 2 s EEG windows, and their DFA exponents *H*_*BB*_ are significantly different from the DFA exponents of white noise obtained by randomly shuffling samples from the same EEG windows using the surrogate test as suggested in Delignieres et al. (2006) and Hausdorff et al. (1995).

##### 2.4.1.2. Determination of Long-Range vs. Short-Range Dependence in the Broadband EEG

After establishing significant correlations in broadband EEG, we identified whether these correlations are short-range or long-range dependence by comparing the fit of corresponding ARMA(*p, q*) and ARFIMA(*p, d, q*) models to each 2 s EEG windows (Wairagkar et al., 2019) using Akaike's information criterion (AIC) (Wagenmakers et al., 2004; Delignieres et al., 2006; Clauset et al., 2009). We first estimated the orders *p* and *q* of ARFIMA and ARMA independently by comparing models of orders *p*=1.10 and *q* = 0 [this range was selected by observing the autocorrelation function and partial autocorrelation function of the EEG (Wairagkar et al., 2019) using AIC]. The ARMA model was estimated using the functions provided by the Econometrics toolbox in MATLAB (https://www.mathworks.com/help/econ/). For estimating ARFIMA, we first fractionally differentiated our EEG window with *d* = *H*_*BB*_ − 0.5 and then fitted ARMA(*p, q*) to it. Then, for each 2 s EEG window of each trial of each participant, AIC was computed to compare ARMA and ARFIMA models of estimated orders. Percentage of total 2 s EEG that showed better fit to ARFIMA model (indicating long-range dependence) than ARMA (indicating short-range dependence) was computed.

##### 2.4.1.3. Identification of LRTC by Validation of Broadband DFA Scaling Exponent *H*_*BB*_ Using Maximum Likelihood DFA

After establishing the long-range dependence, we then narrowed down the type of long-range dependence. If the fluctuations in log–log DFA plot at different timescales follow a linear relationship, then this regularity can be captured by the least squared fit, and the slope of this linear fit represents a well-defined power-law scaling exponent (Botcharova et al., 2013, 2015). We used the maximum likelihood DFA (ML-DFA; Botcharova et al., 2013, 2015) method to show that the linear fit is the best fitting model to the log–log DFA fluctuation plot.

The DFA scaling exponents are valid if the linear model fitted best to the log–log DFA fluctuation plot (Botcharova et al., 2014). First, we assessed the quality of the linear fit using *R*^2^ measure (Linkenkaer-Hansen et al., 2001). Identifying the power-law is inherently difficult (Clauset et al., 2009). Frequently used *R*^2^ measure is insensitive (Botcharova et al., 2013) because it may yield high values even for a non-linear relationship in the data (Clauset et al., 2009); therefore, it is not sufficient to assess the quality of the linear fit. Hence, we used the ML-DFA (Botcharova et al., 2013, 2015) to compare the fits of different models.

We fitted polynomials of order 1–5, an exponential function, a logarithmic function, and a root function as suggested in Botcharova et al. (2013) to the log–log DFA fluctuations and compared them using AIC and Bayesian information criterion (BIC). If the resulting best-fitting model is linear, then we interpret it as an indicator of potential power-law and LRTC.

### 2.5. LRTC in Alpha Envelope and Broadband Stitched EEG During Different Motor Tasks

Traditionally, LRTCs are found in alpha amplitude fluctuations (Linkenkaer-Hansen et al., 2001, 2004; Nikulin and Brismar, 2005; Zhigalov et al., 2016). Since the alpha amplitude has a low frequency, LRTC cannot be computed reliably in short timescales within 2 s windows and require longer timescales. We bandpass filtered each EEG trial between 8 and 13 Hz and segmented it in 2 s sliding windows, then obtained their amplitude envelope by computing the analytic signal using Hilbert transform. We then stitched the corresponding EEG envelope windows from all the trials for each participant to obtain longer EEG segments. For our finger tap dataset with 40 trials per participant, the individual stitched EEG segment was 80 s, and for the EEGMMI dataset with 22 trials per participant, the individual stitched EEG segment was 44 s. Stitching of the data with the same properties does not affect DFA scaling exponent (Hu et al., 2001; Chen et al., 2002; Botcharova et al., 2015). We assume that the corresponding EEG windows from all time-locked trials at each time point during the motor task have same properties, thus ensuring that the stitching will not affect the DFA exponent. We selected the timescales between [2, 20 s] corresponding to approximately box sizes of [2^8^, 2^11^] samples for the finger tap dataset and between [2, 8 s] corresponding to box sizes of [2^8^, 2^10^] for EEGMMI dataset for performing DFA. Then we applied the DFA analysis to obtain scaling exponents *H*_*alpha*_ and validated them using ML-DFA. We also computed DFA exponents on the broadband EEG to verify our single-trial broadband LRTC results.

### 2.6. Classification of Single-Trial Broadband LRTC to Detect Movement and Motor Imagery

We used DFA exponents *H*_*BB*_ from channels C3, Cz, and C4 as three features of single trial broadband LRTC to continuously classify motor task vs. resting state throughout the course of EEG trial using single windows for binary classification of single finger tap and continuous fist movement, feet movement, fist motor imagery, and feet motor imagery from the two datasets.

For our single finger tap dataset, the *H*_*BB*_ feature vectors of each participant were classified into right tap vs. resting state and left tap vs. resting state independently using binary linear discriminant analysis (LDA) classifier. A separate LDA classifier was trained for each sliding window with the feature vectors from corresponding windows in all the movement trials and the same number of feature vectors randomly chosen from the resting state trials of that participant. Each LDA had 40 data samples with three features for each class. A 10 × 10 fold cross-validation was used to obtain the classification accuracies and F1 scores at the time points given by the 2 s sliding windows. The 95% confidence level for binary classification (tap or rest) was obtained from the binomial distribution with *n*= number of EEG trials and *p* = 0.05.

For the EEGMMI dataset, to perform binary classification of each movement/imagery task vs. resting state, we again used the LDA classifier with the single trial *H*_*BB*_ from C3, Cz, and C4 as features. Since there were not enough trials of each condition per participant (22 trials) to train the classifier for each participant individually as above, we used the leave-one-participant-out cross-validation to train the LDA classifier. Leave-one-participant-out scheme gives participant-independent classifier performance on unseen data and is commonly used by several EEG and BCI studies such as Kwon and Im (2021) and Wu et al. (2018). Thus, for each of the participants, a separate LDA classifier was trained for each sliding window as above with the feature vectors from corresponding windows from all the trials from the remaining 108 participants. The classification accuracies and F1 scores were computed along with a 95% confidence level using the binomial distribution.

#### 2.6.1. Statistical Analysis

We used parametric *t*-test and non-parametric Mann–Whitney *U*-test for identifying the statistical significance of normally distributed data and data without normal distribution, respectively, throughout this paper. We determined the normality of the data using the one-sample Kolmogorov–Smirnov test.

## 3. Results

We have identified the temporal dynamics of long-range dependencies in broadband EEG during different types of movements and motor imagery with different paradigms and compared them with the corresponding temporal dynamics of alpha oscillation amplitude envelope fluctuations in the following sections.

### 3.1. Changes in the Broadband LRTC in Single Trials During Different Motor Tasks

**Figure 2:**
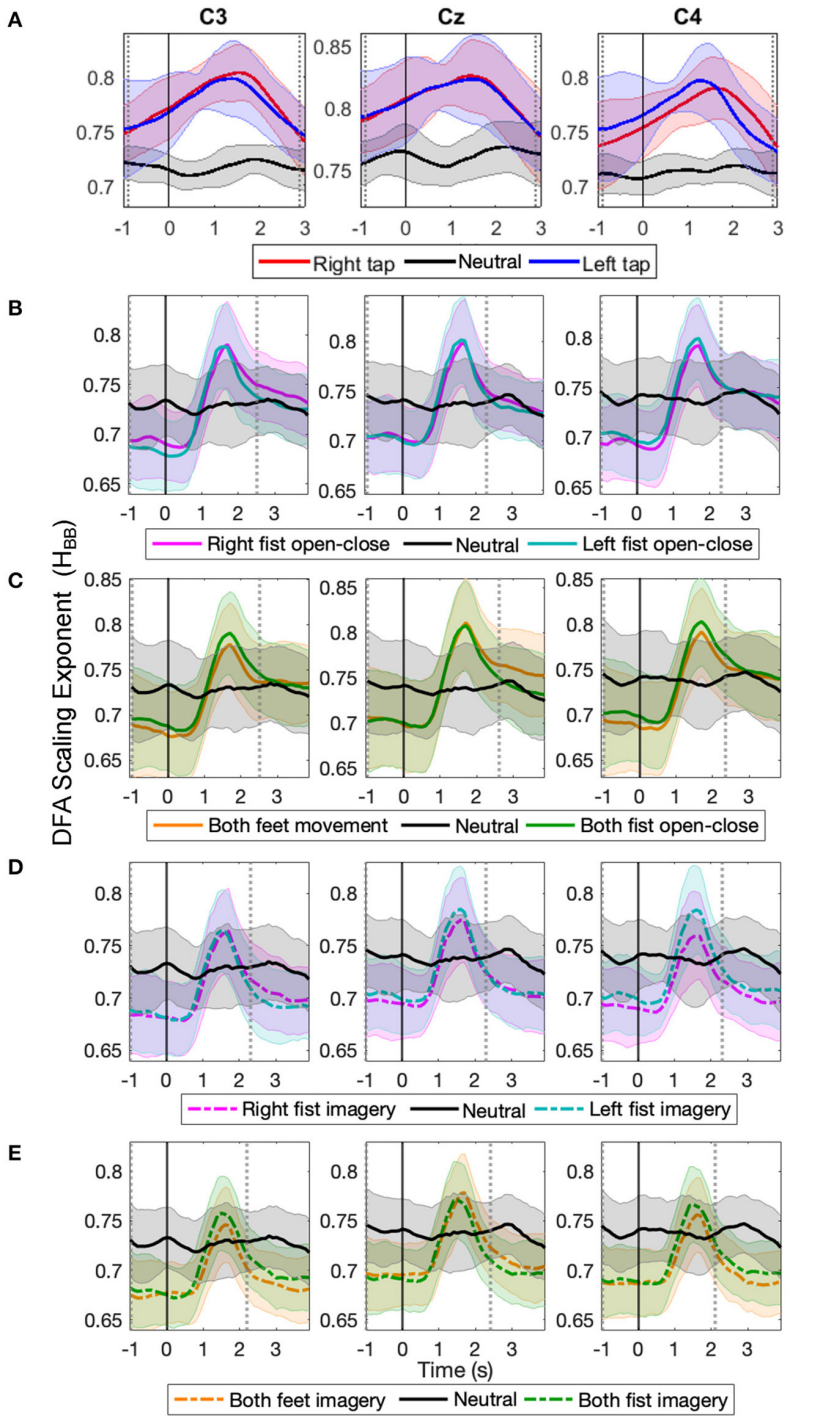
The time evolution of grand average detrended fluctuation analysis (DFA) scaling exponents of broadband electroencephalography (EEG) (*H*_*BB*_) in C3, Cz, and C4 during different movement and motor imagery tasks. The progression of the grand average of mean *H*_*BB*_ of all participants during **(A)** single asynchronous right (red) and left (blue) finger tap and resting state (black), **(B)** continuous right (magenta) and left (cyan) fist open-close movement, **(C)** continuous both feet (orange) and both fist (green) open-close movement, **(D)** motor imagery of continuous right (dashed magenta) and left (dashed cyan) fist open-close, **(E)** motor imagery of continuous both feet (dashed orange) and both fist (dashed green) open-close. Shaded areas show the standard deviation. Solid vertical line at 0 s marks finger tap onset in **(A)** and motor task cue in **(B–E)**. Dotted gray vertical lines show the period in which *H*_*BB*_ of motor task trials is significantly different (*p* < 0.05, Mann–Whitney *U*-test) from that of resting state trials. The *H*_*BB*_ shows clear increase during all motor tasks.

The time evolution of the grand average *H*_*BB*_ obtained on a single trial basis in C3, Cz, and C4 are shown in Figure 2 for 14 participants' right and left self-initiated asynchronous single finger tap (Figure 2A, for individual participants LRTC, see Supplementary Figure 1) and 109 participants' cued continuous tasks of right and left fist open-close movement (Figure 2B), both feet and fist open-close movement (Figure 2C), motor imagery of right and left fist open-close (Figure 2D), and motor imagery of both feet and fist open-close (Figure 2E). A clear increase in *H*_*BB*_ is seen during all motor tasks.

For the finger tap dataset, the *H*_*BB*_ increased during movement intention and execution of right and left index finger single tap and restored to its baseline level afterwards. For the EEGMMI dataset, the *H*_*BB*_ also increased during movement and imagery shortly after the cue and then restored to its baseline levels before the continuous motor task was over (the continuous motor task in this dataset lasted from 0 to 4 s). There was no such increase in the *H*_*BB*_ during the resting state in both the datasets. The *H*_*BB*_ during the motor task and resting state was between 0.5 and 1, indicating the presence of power-law decay and LRTC in the autocorrelation of broadband EEG (Berthouze and Farmer, 2012). The long-range temporal correlations in the EEG became stronger during voluntary movement and motor imagery.

The solid vertical line at 0 s marks the onset of the self-initiated asynchronous single finger-tap in Figure 2A, and it marks the start of cue for the continuous motor task that lasts for the next 4 s in the EEGMMI dataset in Figures 2B–E. The two vertical dotted gray lines show the period between which *H*_*BB*_ for motor tasks colored traces is significantly different from the resting state (black) (*p* < 0.05, Mann–Whitney *U*-test, *n*= total number of trials in all participants of each dataset). The peaks of *H*_*BB*_ in the individual trials were not time-locked or aligned. Since the EEGMMI dataset had more participants, the standard deviation of *H*_*BB*_ is also larger, as indicated by the shaded area. There is no visible difference in the grand average LRTCs of different tasks in the EEGMMI database. Being able to compute the DFA exponent on a single-trial basis that shows significant changes during a range of motor execution and imagery tasks with different paradigms can allow its use as a feature for motor-based BCIs. In the case of self-initiated voluntary movements, LRTCs can also predict movement before its onset, as seen from finger tap LRTCs in Figure 2A.

Before validating broadband LRTC, we validated raw EEG by computing spectrograms (see Supplementary Figure 2). A clear attenuation of alpha band power around 10 Hz was observed, which indicates the presence of ERD. Thus, the presence of ERD, a commonly used correlated of movement validates EEG data by confirming that it contains motor-related information. Details of the relationship between ERD and broadband LRTC are given in Wairagkar et al. (2019).

#### 3.1.1. Validation of Broadband LRTC

##### 3.1.1.1. Identification of Significant Correlations in Broadband EEG Using Surrogate Test

The surrogate test confirmed that *H*_*BB*_ in 2 s EEG windows were significantly different from the DFA exponents of randomly shuffled samples from the same EEG windows (*p* = 0, Mann–Whitney *U*-test, *n*= individual windows in all the participants). The scaling exponents of the shuffled data were close to 0.5, as shown in Supplementary Figure 3, confirming the presence of white noise with no correlations. Thus, there were significant correlations present in the broadband 2 s EEG.

##### 3.1.1.2. Determination of Long-Range vs. Short-Range Dependence in the Broadband EEG

The comparison of ARMA for short-range dependence and ARFIMA for long-range dependence using AIC resulted in the selection of the ARFIMA model by AIC for about 80% of the total 2 s EEG windows compared. Hence, the ARFIMA model was a better fit for the short broadband EEG windows confirming that the long-range dependence was indeed present.

##### 3.1.1.3. Identification of LRTC by Validation of Broadband DFA Scaling Exponent *H*_*BB*_ Using Maximum Likelihood DFA

The average *R*^2^ measure for all the EEG windows of all the participants in all the channels was 0.96±0.02 (mean ± SD), indicating that the regression line of the DFA fluctuation plot is a close fit. The ML-DFA method resulted in the selection of the linear model for 80% of the times in all the windows across all the trials, channels, and participants during all the conditions. In the remaining cases, a quadratic polynomial was chosen. We attribute this to the noise induced in computing the root mean square DFA fluctuations at the larger timescales due to short EEG segments. The distribution of the coefficient of the linear term in the linear model and the quadratic model was the same when these respective models were selected as best fitting. In the case where the quadratic model was chosen, the ratio of the coefficient of the quadratic term to that of the linear term was small (0.02), showing a significantly smaller contribution of the quadratic term than the linear term (*p* < 0.05, two-tailed *t*-Test, *n*=individual windows in all the participants) and hence we did not discard these EEG windows. All these factors led us to conclude that the log–log DFA fluctuation plots were linear and the *H*_*BB*_ was indeed valid.

### 3.2. Changes in LRTC in Alpha Envelope and Broadband Stitched EEG During Different Motor Tasks

We obtained LRTC on longer stitched EEG segments in both broadband and alpha oscillation amplitude envelope during different motor execution and imagery tasks from both datasets. We then compared the changes in the broadband LRTC and alpha amplitude envelope LRTC in the following sections.

#### 3.2.1. Changes in Alpha Envelope LRTC (*H*_*alpha*_) Using Stitched EEG

**Figure 3:**
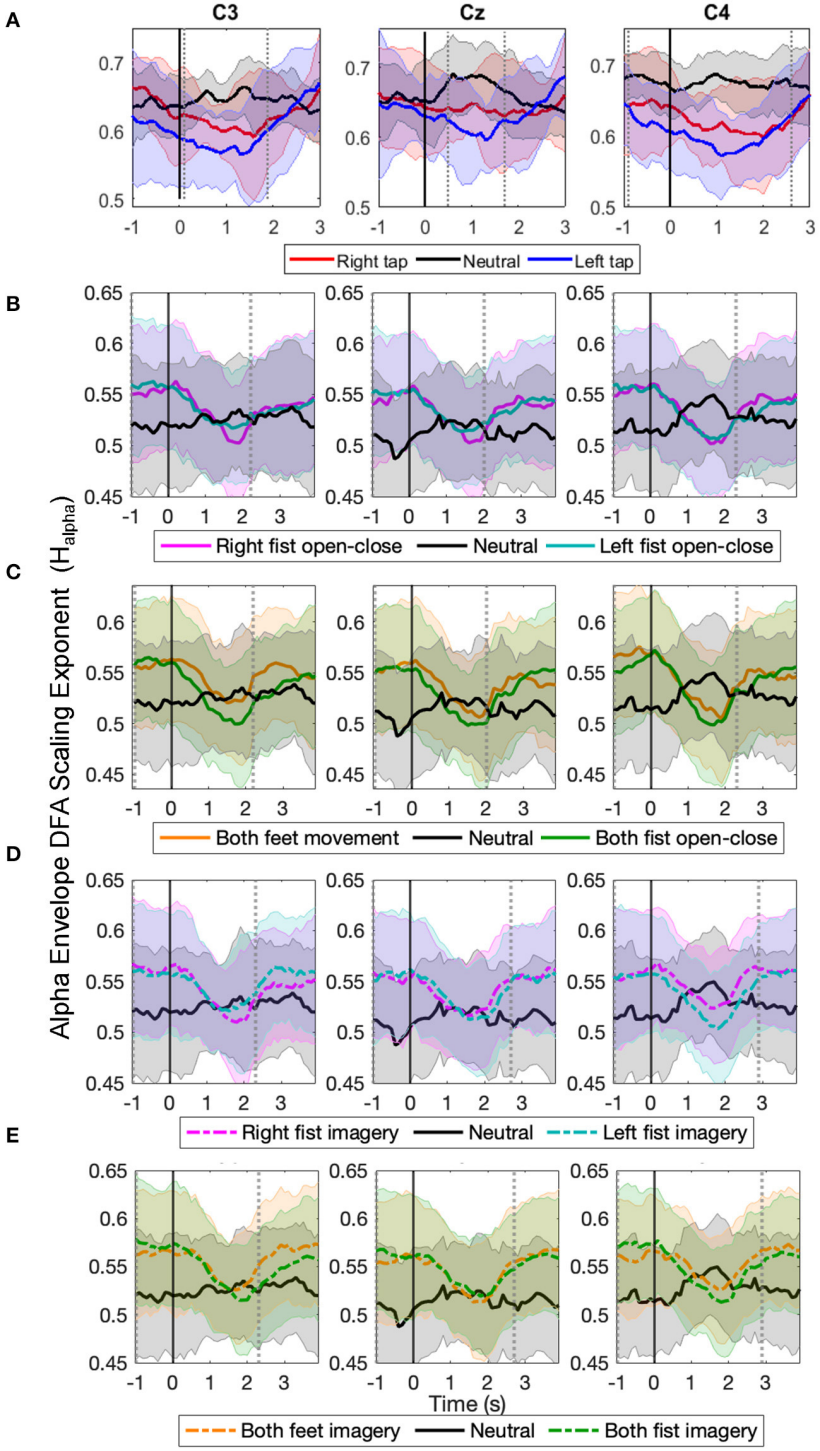
The time evolution of grand average detrended fluctuation analysis (DFA) scaling exponents in the envelope of alpha oscillations of stitched electroencephalography (EEG) (*H*_*alpha*_) in C3, Cz, and C4 during different movement and motor imagery tasks. The progression of the grand average of alpha oscillation amplitude envelope DFA scaling exponent *H*_*alpha*_ of all participants during **(A)** single asynchronous right (red) and left (blue) finger tap and resting state (black), **(B)** continuous right (magenta) and left (cyan) fist open-close movement, **(C)** continuous both feet (orange) and both fist (green) open-close movement, **(D)** motor imagery of continuous right (dashed magenta) and left (dashed cyan) fist open-close, **(E)** motor imagery of continuous both feet (dashed orange) and both fist (dashed green) open-close. Shaded areas show the standard deviation. Solid vertical line at 0 s marks finger tap onset in **(A)** and motor task cue in **(B–E)**. Dotted gray vertical lines show the period in which *H*_*alpha*_ of motor task trials is significantly different (*p* < 0.05, Mann–Whitney *U*-test) from that of resting state trials. The *H*_*alpha*_ shows clear decrease during all motor tasks.

The time evolution of the grand average alpha envelope LRTCs in Figure 3 shows that *H*_*alpha*_ values decreased significantly (*p* < 0.05, Mann–Whitney *U*-test, *n* = number of participants) during all the motor execution consistently with the literature (Linkenkaer-Hansen et al., 2004) and during all motor imagery tasks as well. The decrease in alpha envelope LRTC is in contrast to the increase in the broadband LRTC of the same motor tasks (Figure 2), indicating that different long-range dependent processes coexist during a motor task. The decrease in the alpha envelope LRTC was observed in the single asynchronous finger tap task from our finger tapping dataset (Figure 3A) and in cued continuous tasks of right and left fist open-close movement (Figure 3B), both feet and fist open-close movement (Figure 3C), motor imagery of right and left fist open-close (Figure 3D), and motor imagery of both feet and fist open-close (Figure 3E) from the EEGMMI dataset. The decrease in *H*_*alpha*_ is prominent in C3 and C4. We validated these DFA exponents from stitched EEG, which ranged between 0.5 and 1 using ML-DFA, and confirmed the presence of LRTCs in the fluctuations of the alpha amplitude envelope of stitched EEG.

#### 3.2.2. Verification of Changes in Broadband LRTC (*H*_*BB*_) During Movement Using Stitched EEG

**Figure 4:**
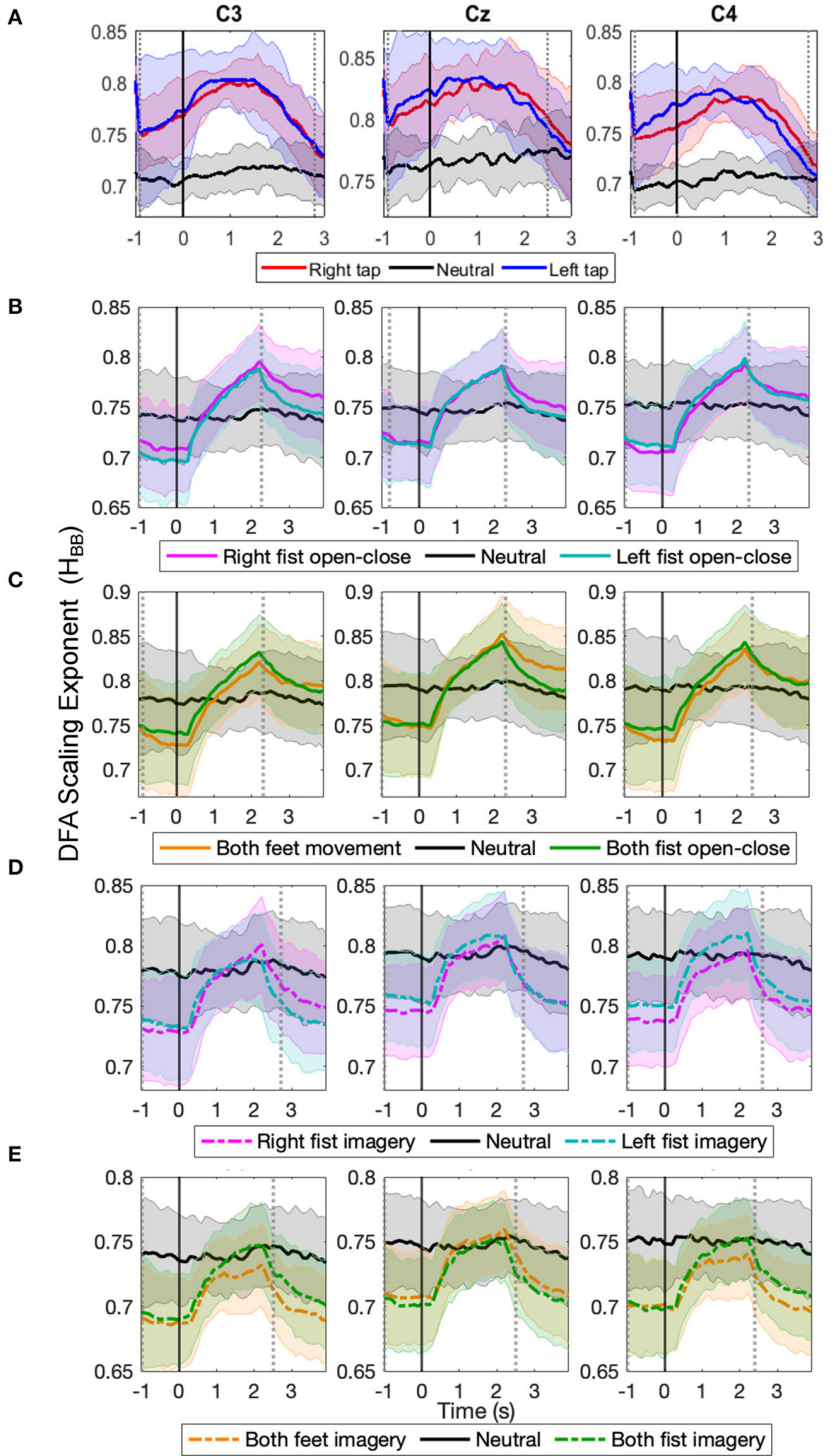
The time evolution of grand average detrended fluctuation analysis (DFA) scaling exponents in broadband stitched electroencephalography (EEG) (*H*_*BB*_) in C3, Cz, and C4 during different movement and motor imagery tasks. The progression of the grand average of broadband DFA scaling exponent *H*_*BB*_ from stitched EEG segments of all participants during **(A)** single asynchronous right (red) and left (blue) finger tap and resting state (black), **(B)** continuous right (magenta) and left (cyan) fist open-close movement, **(C)** continuous both feet (orange) and both fist (green) open-close movement, **(D)** motor imagery of continuous right (dashed magenta) and left (dashed cyan) fist open-close, **(E)** motor imagery of continuous both feet (dashed orange) and both fist (dashed green) open-close. Shaded areas show the standard deviation. Solid vertical line at 0 s marks finger tap onset in **(A)** and motor task cue in **(B–E)**. Dotted gray vertical lines show the period in which *H*_*BB*_ of motor task trials is significantly different (*p* < 0.05, Mann–Whitney *U*-test) from that of resting state trials. The *H*_*BB*_ shows clear increase during all motor tasks.

The progression of the broadband LRTC (*H*_*BB*_) of stitched EEG shown in Figures 4A–E also shows that the scaling exponents increase significantly during different motor execution and imagery tasks from our finger tap dataset and EEGMMI dataset (*p* < 0.05, Mann–Whitney *U*-test, *n*=number of participants). This is consistent with the changes in the single trial broadband EEG from Figure 2. The *H*_*BB*_ values are similar for both 2 s windows and stitched EEG and are in the range of 0.5–1. The scaling exponents of the stitched EEG were also validated using ML-DFA. The linear model was selected by AIC and BIC individually for 96% times of all the stitched EEG segments in all the windows of all the three channels in all the participants and all conditions. This confirmed the validity of the *H*_*BB*_ estimates on single 2 s windows and the presence of LRTC in the broadband EEG. ML-DFA results showed that the quadratic model was previously incorrectly selected in the single-trial DFA because of the noise in the estimation of RMS fluctuations at higher timescales due to short EEG segments.

#### 3.2.3. Correlation Between Broadband LRTC (*H*_*BB*_) and Alpha Envelope LRTC (*H*_*alpha*_)

The broadband LRTC and alpha envelope LRTC show changes in opposite directions during all the motor tasks. The broadband LRTCs increased (Figures 2, 4), while the alpha envelope LRTCs decreased (Figure 3) during same motor tasks. The increase in broadband LRTC and the decrease in the corresponding alpha envelop LRTC are inversely correlated and temporally co-evolve during a motor task. However, in the resting state, broadband and alpha envelope LRTC are uncorrelated. This distinction in the behavior of the two LRTC dynamics during movement and in the resting state is shown in Supplementary Figure 4 by the scatter plot between *H*_*BB*_ and *H*_*alpha*_ of stitched EEG during right and left finger tap and resting state, and their corresponding correlation coefficients.

#### 3.2.4. Timescales of Broadband LRTCs and Alpha Envelope LRTCs

**Figure 5:**
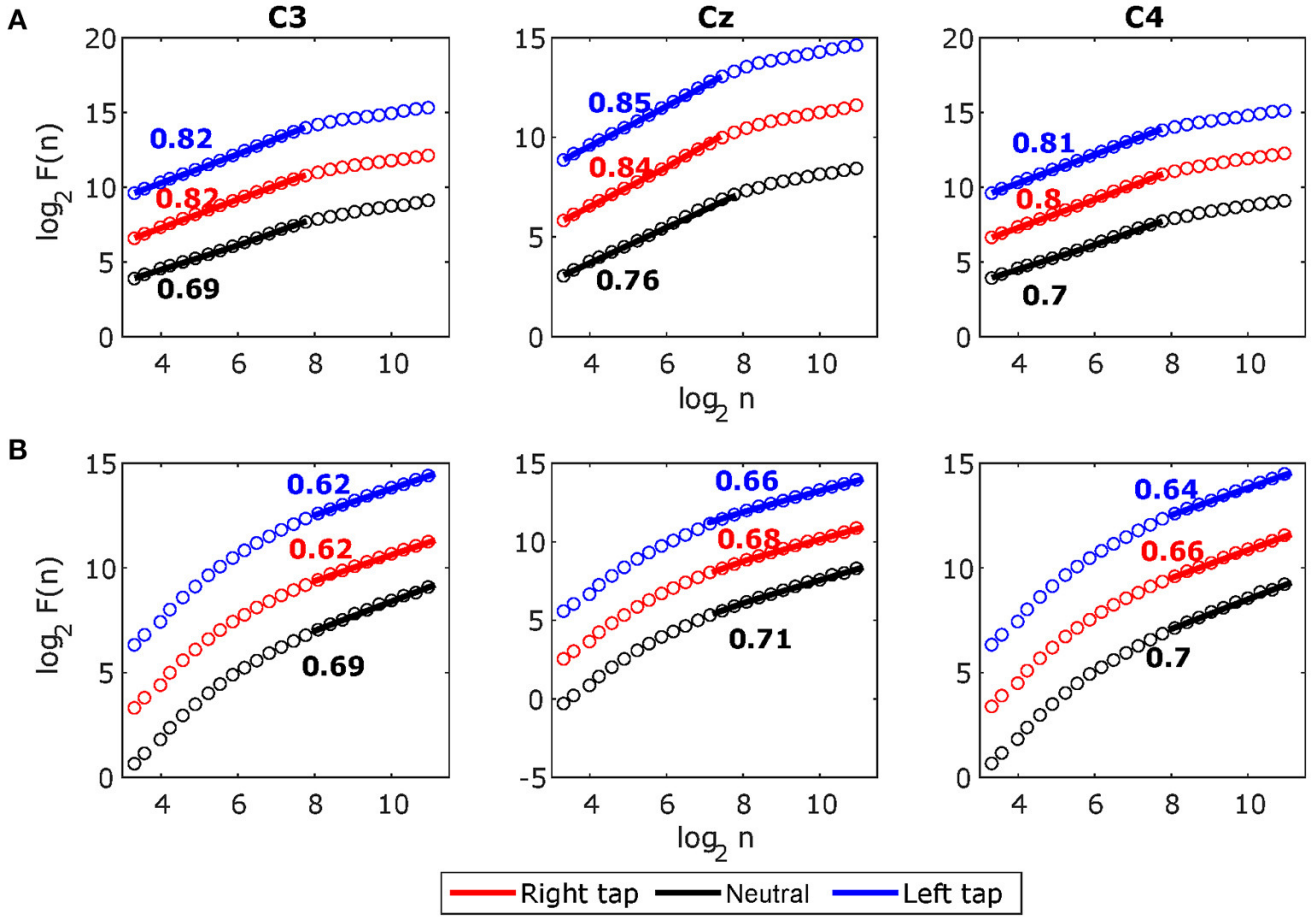
The grand average detrended fluctuation analysis (DFA) plots for stitched broadband electroencephalography (EEG) and alpha amplitude fluctuations during movement in C3, Cz, and C4. **(A)** The grand average DFA plots for stitched broadband EEG during right tap (red), left tap (blue), and resting state (black) for all the participants. This EEG segment is extracted from −1 to +1 s of the movement onset. The Hurst exponents *H*_*BB*_ obtained from the slope of the fitted line to the DFA plots are shown. **(B)** The grand average DFA plots for stitched alpha amplitude fluctuations *H*_*alpha*_. The broadband DFA exponents are valid over shorter scales as there is a crossover point at 8 (i.e., *log*_2_ of 256 samples) and the alpha envelope DFA exponents are valid over longer timescales. With sampling frequency of 128 Hz, smallest DFA box size of 10 samples (*log*_2_10 = 3.32) correspond to 78 ms, crossover point at 256 samples (*log*_2_256 = 8) correspond to 2 s, and largest DFA box size of 2,241 samples (*log*_2_2241 = 11.13) correspond to 17.5 s.

Figure 5A shows the grand average broadband DFA plots, and Figure 5B shows the grand average alpha envelope DFA plots. The broadband DFA fluctuations are linear (and thus the scaling exponent *H*_*BB*_ is valid) in the log–log plot on the shorter timescales < 2^8^, while the alpha envelope DFA fluctuations are linear only on the longer timescales > 2^8^. The maximum timescale of < 2^8^ corresponds to 2 s on which the broadband DFA fluctuations are linear (i.e., the maximum possible box size can be 2 s for broadband DFA if sufficiently long EEG segment is available), however, the slope of the DFA fluctuations remains the same on shorter timescales and thus broadband DFA scaling exponent *H*_*BB*_ can be accurately estimated from shorter EEG segments. This shows that broadband LRTCs are present on the shorter timescales capturing faster changes in the dynamics and alpha envelope LRTCs are present on the longer timescales representing slower changes in the dynamics. Though both the LRTCs show change in dynamics during different motor tasks, broadband LRTCs can be used to identify these change almost instantly as opposed to the alpha envelope LRTCs, which require longer EEG segments for detection.

### 3.3. Classification Accuracies of Single-Trial Broadband LRTC to Detect Movement and Motor Imagery

Table 1 shows the grand average of peak binary classification accuracies of different motor tasks vs. resting state and their F1 scores. Maximum classification accuracy of 76.07±6.4% was obtained for finger tap. Classification accuracies for different motor execution and imagery tasks from the EEGMMI dataset were similar to that of the finger tapping dataset. Peak classification accuracy for single finger tap was obtained around 1 s after the movement onset, which corresponds to the EEG window from −1 to +1 s, and peak classification accuracies were obtained around the same time for motor execution and imagery tasks from the EEGMMI database as well. This time of peak accuracies corresponds to the time of the maximum difference between a motor task and resting state *H*_*BB*_, which was also approximately at 1–1.5 s (see Figure 2). However, all classification accuracies crossed the significance threshold (*p* < 0.05) of chance level earlier than this. In the case of a self-initiated asynchronous single finger tap, the time of movement intention was recorded as the time at which the classification accuracy crosses the significance threshold. We observed that finger tap movement can be predicted on average 0.5 s before its actual onset using the LRTCs in broadband EEG over shorter timescales.

**Table 1:** The grand average of peak linear discriminant analysis (LDA) classification accuracies for different motor tasks vs. resting state of all the participants using single trial broadband long-range temporal correlation (LRTC) scaling exponents from C3, Cz, and C4 as features.

	**Average classification accuracy (%)**	**F1 score**
Right finger tap vs. neutral	76.07 (6.40)	0.76 (0.06)
Left finger tap vs. neutral	75.69 (6.77)	0.75 (0.07)
Right fist open-close vs. neutral	73.00 (8.84)	0.79 (0.08)
Left fist open-close vs. neutral	72.24 (8.50)	0.79 (0.08)
Both feet movement vs. neutral	74.41 (9.67)	0.80 (0.08)
Both fist open-close vs. neutral	73.26 (9.09)	0.80 (0.08)
Right fist imagery vs. neutral	70.54 (10.03)	0.77 (0.09)
Left fist imagery vs. neutral	72.60 (9.08)	0.79 (0.08)
Both feet imagery vs. neutral	71.51 (9.87)	0.78 (0.09)
Both fist imagery vs. neutral	73.18 (10.18)	0.80 (0.09)

*Standard deviations are given in brackets. All values are significantly above chance level (p < 0.05)*.

## 4. Discussion

The temporal dynamics of broadband EEG during voluntary movement remain mostly unexplored in the literature as opposed to the commonly studied narrow band oscillatory ERD/S (Yuan and He, 2014) or slow potentials of MRCP (Bai et al., 2011; Ibáñez et al., 2014). In our previous work, we have shown that EEG is composed of coexisting broadband short- and long-range temporal correlations, which we modeled with ARFIMA, and both these processes show significant changes during finger tap movement intention and execution (Wairagkar et al., 2019). Building upon our previous work, to deepen our understanding of LRTCs during different motor tasks, we investigated the nature of changes in LRTCs further in broadband and alpha envelope EEG in this study and made the following novel contributions: (1) We demonstrated wider applications of broadband LRTC with consistent changes over different motor tasks by using two independent datasets comprising a total of 123 participants with five different motor execution and imagery tasks recorded using two different experimental paradigms (asynchronous single finger tap and cued continuous movements), thus enhancing its usability. (2) We systematically validated the presence of single trial broadband LRTC on short timescales. This ubiquitous presence of broadband LRTC in different motor tasks suggests existence of potential power-law dynamics in the temporal broadband brain activity as well. (3) We observed contrasting behavior of LRTC dynamics in broadband and alpha envelope. Broadband LRTCs increased during motor tasks (Figure 2). In contrast, LRTCs in the narrow band alpha oscillation envelop decreased in corresponding tasks (Figure 3), consistent with the literature (Botcharova et al., 2015). Thus, two distinct fast and slow LRTC processes coexist in arrhythmic broadband EEG and oscillatory alpha wave envelop, respectively. (4) For the first time, we showed LRTC dynamics (of broadband EEG and alpha envelope) during motor imagery, which has never been studied before. (5) Single trial broadband LRTC can be used independently as a neural correlate of different motor execution and imagery tasks for robust classification.

We systematically validated LRTCs in the short 2 s broadband EEG windows by establishing first that there is a significant correlation in the EEG using surrogate test (Hausdorff et al., 1995), then identifying the nature of these correlations as long-range dependence using ARFIMA modeling (Wagenmakers et al., 2004; Torre et al., 2007; Wairagkar et al., 2019) and finally showing that these long-range dependencies are in fact LRTCs (power-law decay of autocorrelation) using ML-DFA (Botcharova et al., 2013). Clauset et al. (2009) discuss that identifying power-law is a difficult problem and linearity on the log-log plot is a necessary but not sufficient condition; the best approach for identifying the power-law is by comparing different models and determining whether the power-law is the best fitting model (which we implemented with ML-DFA). Here, we accept the assumption that the linear trend of the DFA fluctuations at different scales is an indicator of the power-law according to several studies in EEG (Linkenkaer-Hansen et al., 2001; Nikulin and Brismar, 2005; Botcharova et al., 2014). Thus, we rigorously validate the presence of LRTCs in broadband EEG in line with this suggested approach. The broadband EEG, which is non-oscillatory and arrhythmic and hence scale-free and stochastic, coexists with oscillatory processes in the brain (He, 2014). Hence, we can obtain a good estimate of the Hurst exponent (*H*_*BB*_, Figure 2) using short segment (2 s) of broadband EEG with 256 samples (Delignieres et al., 2006), which we also verified using the longer stitched EEG segments (Figure 4). Thus, our results prove the presence of this scale-free property of the broadband EEG over small timescales (from 78 ms up to 2 s; Figure 5).

According to Kantelhardt (2009), the power-law is valid if it exists for at least one order of magnitude, which we obtain in the stitched broadband EEG (78 ms–2 s). The LRTC in the single-trial 2 s broadband EEG was present over a short range of 78 ms–0.5 s. The analysis of stitched broadband EEG allowed us to extrapolate that these LRTC dynamics hold up to 2 s (Figure 5). The range of 78 ms–0.5 s for single trials falls within the recommended range for the DFA plot of filtered data by Li et al. (2018) to avoid the effects of filtering on DFA. The study by Hu et al. (2013) also found LRTCs in the neuronal firing in a similar range of timescales as ours, which helps in confirming that LRTCs exist in the shorter timescales in the neuronal activity.

Broadband LRTC was present only in shorter timescales irrespective of the length of the EEG, and it did not extend over longer timescales (Figure 5A). The stitched *H*_*BB*_ DFA plots in Figure 5A show that there is a crossover at 2 s (2^8^). This suggests that the broadband activity may be multifractal (Kantelhardt et al., 2002), in which a single power-law is valid in the range of 78 ms–2 s, beyond which there may be a different scale-free trend with a different scaling exponent. The investigation of multifractality in broadband EEG, which is often modeled by stochastic processes, will be an interesting future avenue as we have shown in our previous work that arrhythmic EEG can be modeled by stochastic ARFIMA (Wairagkar et al., 2019); however, it is beyond the scope of this paper since we do not observe these longer timescales in single short 2 s EEG windows, which are essential for application in BCI. Single trial EEG analysis not only plays an important role in BCI but also in cognitive neuroscience to study the ongoing instantaneous changes in temporal dynamics during memory task (Ratcliff et al., 2016), cognitive functions (Debener et al., 2006), and voluntary responses (Yamanaka and Yamamoto, 2010). Single trial broadband LRTC could have potential applications in studying instantaneous dynamics of other cognitive tasks as well.

The range of timescales over which *H*_*BB*_ is valid is especially interesting because it enabled monitoring of the instantaneous modulations in the broadband LRTC, facilitating the detection of movements and motor imagery in single trials. These LRTCs over short timescales characterize long-memory of the faster processes as opposed to longer timescales that contain the long-memory of slower brain processes. In the case of a motor task, the brain must switch between different cortical areas and modulate the neuronal activity selectively to produce dynamic movement; stronger LRTCs may provide favorable conditions for this (Samek et al., 2016). This might be one of the reasons for the increase in broadband LRTCs during a motor task. We infer that the broadband activity may be multifractal and more dynamic with changes happening over shorter timescales. Multifractality was also observed by Hu et al. (2013) in neuronal firing during a movement task. Consistent with our results, they also observed that the LRTCs increased during the reaching movement in neuronal firings, which correlated with the movement trajectory, and the LRTC was reset at the beginning of the next movement. There may be several different mechanisms giving rise to the power-law dynamics (Stumpf and Porter, 2012) in brain activity. Further investigation is needed to identify the mechanisms and causes for the increase in the broadband LRTC during motor tasks.

Since ERD is the most common measure of identifying movement from EEG in literature, the question arises about the relationship between ERD and our proposed broadband LRTC. Linkenkaer-Hansen et al. (2001) have specified that the power spectrum and LRTC are not equivalent. We have shown in Wairagkar et al. (2019) that ERD and broadband LRTC are indeed complementary processes and provide different information about a motor task. Removing the broadband LRTC from EEG by fractional differencing does not affect the ERD output, and removing ERD from EEG by filtering out the ERD frequency band does not affect the broadband LRTC. Becker et al. (2018) reported that the increase in power of alpha oscillations in the spontaneous activity caused a decrease in the long-range dependence in the lower frequencies (<5 Hz) of EEG. Extending their finding to a wider range of broadband frequencies in EEG, we also obtained this inverse relationship between alpha power (which decreases leading to ERD (Pfurtscheller and Lopes Da Silva, 1999) and broadband LRTC, which increased during different motor tasks. However, Becker et al. (2018) concluded the existence of this causality merely by identifying the lag at which the maximum correlation between the alpha power and LRTCs in < 5 Hz band occurs, which may not be sufficient to establish causality. Moreover, such a causal relationship does not exist in the case of wider broadband LRTC (0.5–45 Hz), which we have shown in our previous work (Wairagkar et al., 2019).

The LRTCs in the EEG are usually detected in the amplitude fluctuations of the narrow band oscillations (Linkenkaer-Hansen et al., 2001; Nikulin and Brismar, 2005; Zhigalov et al., 2015). Such LRTCs of alpha oscillations decrease due to a sensorimotor stimulus or task (Linkenkaer-Hansen et al., 2004). We have also observed the same effect on LRTCs in the amplitude fluctuations of the alpha oscillations obtained from the stitched EEG windows (see Figure 4). Computing LRTCs in the alpha oscillation amplitude requires a longer EEG segment. Thus, LRTC analysis of alpha amplitude faces limitations on observing fast LRTC changes and their continuous assessment in single trials during a short event such as movement, unlike broadband EEG, which renders alpha envelop LRTCs inappropriate for use in BCI. The continuous monitoring of the ongoing changes in broadband LRTC achieved using 2 s sliding windows gives an additional dimension of movement-related information.

All motor tasks caused modulations in the broadband and oscillatory LRTC dynamics, but in opposite directions and over different timescales. The *H*_*BB*_ and *H*_*alpha*_ obtained from stitched EEG are uncorrelated during the resting state, and there is a switch in their behavior during movement when they become coupled and inversely correlated (with a strong average correlation coefficient of −0.8 as shown in Supplementary Figure 4). In the resting state, *H*_*BB*_ has a lower variance, and *H*_*alpha*_ has a broader range of observed values. Hence, the alpha amplitude and broadband LRTCs reflect distinct processes occurring during voluntary movement, capturing the slow processes on the macroscopic level and complementary fast processes on the microscopic level, respectively.

Both finger tapping and EEGMMI datasets showed a similar buildup of broadband LRTC (and a corresponding decrease of alpha envelope LRTC); however, the LRTC peak was narrow in case EEGMMI dataset with cued continuous movement and imagery and the increase in broadband LRTC (and the corresponding decrease in alpha envelope LRTC) started around 0.5 s after the cue (see Figures 4B–E). This could be because of some evoked response to a cue or because of delayed reaction time for movement or imagery initiation by participants in response to the cue. ERD was also delayed by about 0.5 s after the onset of cue in all the motor tasks from this dataset (see Supplementary Figures 2B–E). In the finger tapping dataset, this increase in broadband LRTC starts before the onset of single finger movement (see Figure 4A) because it is also capturing movement intention for initiating the finger tap movement voluntarily. Same effect was observed in ERD of finger tapping dataset where ERD started before the onset of finger tap (see Supplementary Figure 2A). In the EEGMMI dataset continuous motor tasks, the broadband LRTC restores to its baseline level before the continuous motor execution or imagery ends at 4 s. No such return to baseline level is observed in the ERD of this dataset (see Supplementary Figures 2B–E). This could be another indicator that broadband LRTC captures information about movement intention and initiation and differs from the information content of ERD.

We have shown that we can reliably detect different motor execution and imagery tasks using LRTC from 2 s single broadband EEG segments independently with average classification accuracies in the range of 70.54±10.03% to 76.07±6.40% (Table 1). We were also able to predict the single finger tap movement 0.5 s before its onset, which also had the highest classification accuracy of 76.07±6.4%. Slightly lower classification accuracies for continuous fist and feet motor execution and imagery from the EEGMMI dataset can be attributed to the leave-one-participant-out LDA classifier training scheme as for each participant, the classifier was trained with the data from the remaining participants, whereas, for finger tapping dataset, the classifier was trained for each participant using their own data with 10 x 10 fold cross-validation. The classification accuracies using broadband LRTC are comparable to the accuracies obtained in the BCI literature (Ibáñez et al., 2014; Lew et al., 2014; Lopez-Larraz et al., 2014; Xu et al., 2014; Padfield et al., 2019; Zhang et al., 2021). Thus, broadband LRTCs can be used as features independently for application in BCI. In our previous work (Wairagkar et al., 2019), we showed that combining short-range and long-range temporal correlation features increases classification accuracy, thus broadband LRTC being a novel complementary process can be used in combination with motor-related features to obtain high classification accuracies for motor execution or imagery-based BCI. Though we used an offline analysis in this paper, the DFA analysis is done on a single trial basis with movement detected every 100 ms based on the 2 s EEG segment and can be easily adapted for online BCI. The successful application of broadband LRTCs as features for offline BCI serves as a robust method of validation of their dynamical changes occurring during motor tasks. Having broadband LRTC as an additional neural correlate with the capability of detecting movement may also be useful in the cases where individuals are unable to operate BCIs with common ERD and MRCP features.

## 5. Conclusions

We demonstrated by deeper investigation and rigorous validation of changes in the single trial broadband LRTC over short timescales that it is a robust neural correlate of movement, expanding our previous understanding. Broadband LRTC showed consistent changes and is hence generalizable over different motor tasks such as the finger, fist, and feet movements and motor imagery with different experimental paradigms including single self-initiated asynchronous movement and cued continuous motor execution and imagery. We proved the validity and reproducibility of broadband LRTC on short timescales on single trial 2 s EEG segments by applying it to two independent (our own and external) EEG datasets recorded from a total of 123 participants. LRTCs in the broadband EEG increased significantly (*p* < 0.05) during motor tasks (Figures 2, 4). In contrast, LRTCs in the alpha oscillation amplitude envelope (which we could only observe by stitching the EEG windows together) decreased during motor tasks (Figure 3). Thus, there are complementary fast processes from the scale-free broadband arrhythmic neuronal activity and slow processes from oscillatory neuronal activity coexisting and contributing to voluntary movement tasks. We also identified for the first time, changes in LRTC dynamics during motor imagery which has not been explored before in the literature.

The broadband LRTC has proved to be a novel neural correlate that can be used independently to detect different types of movement or imagery vs. resting state every 100 ms on a single trial basis with the classification accuracy in the range of 70.54±10.03% to 76.07±6.40%. It can also predict a single voluntary asynchronous finger tap 0.5 s before its onset. Hence, the broadband LRTC provides a new stream of movement-related information for application in BCI with different paradigms, including single or continuous movement and motor imagery.

## Data Availability Statement

The datasets presented in this study can be found in online repositories. The names of the repository/repositories and accession number(s) can be found at: University of Reading Research Data Archive (http://dx.doi.org/10.17864/1947.117) for the single finger tap EEG dataset that we generated, and PhysioNet (https://doi.org/10.13026/C28G6P) for the EEG Movement/Motor Imagery dataset.

## Ethics Statement

The studies involving human participants were reviewed and approved by the ethics committee of the School of Systems Engineering, University of Reading, UK. The participants provided their written informed consent to participate in this study.

## Author Contributions

MW conceptualized the study, designed and conducted the experiments, developed the methodology, analyzed and interpreted the results, created the visualizations, and wrote the original manuscript. YH and SN supervised the study, conceptualized the methodology, validated and interpreted the results, and reviewed the manuscript. All authors contributed to the article and approved the submitted version.

## Conflict of Interest

The authors declare that the research was conducted in the absence of any commercial or financial relationships that could be construed as a potential conflict of interest.
