# Supplementary material for: Dynamics of Long-Range Temporal Correlations in Broadband EEG During Different Motor Execution and Imagery Tasks
Source: Front Neurosci. 2021 May 28;15:660032. doi: 10.3389/fnins.2021.660032 (PMC8193084; doi:10.3389/fnins.2021.660032)
Supplement: Supplementary file 1 [file Data_Sheet_1.PDF]

# Supplementary Material

## 1 SUPPLEMENTARY FIGURE 1

The time evolution of the mean $H_{BB}$ obtained on a single-trial basis for individual participants in C3, Cz, and C4 for single asynchronous finger tap from our finger tapping dataset (Wairagkar et al. (2017), http://dx.doi.org/10.17864/1947.117)

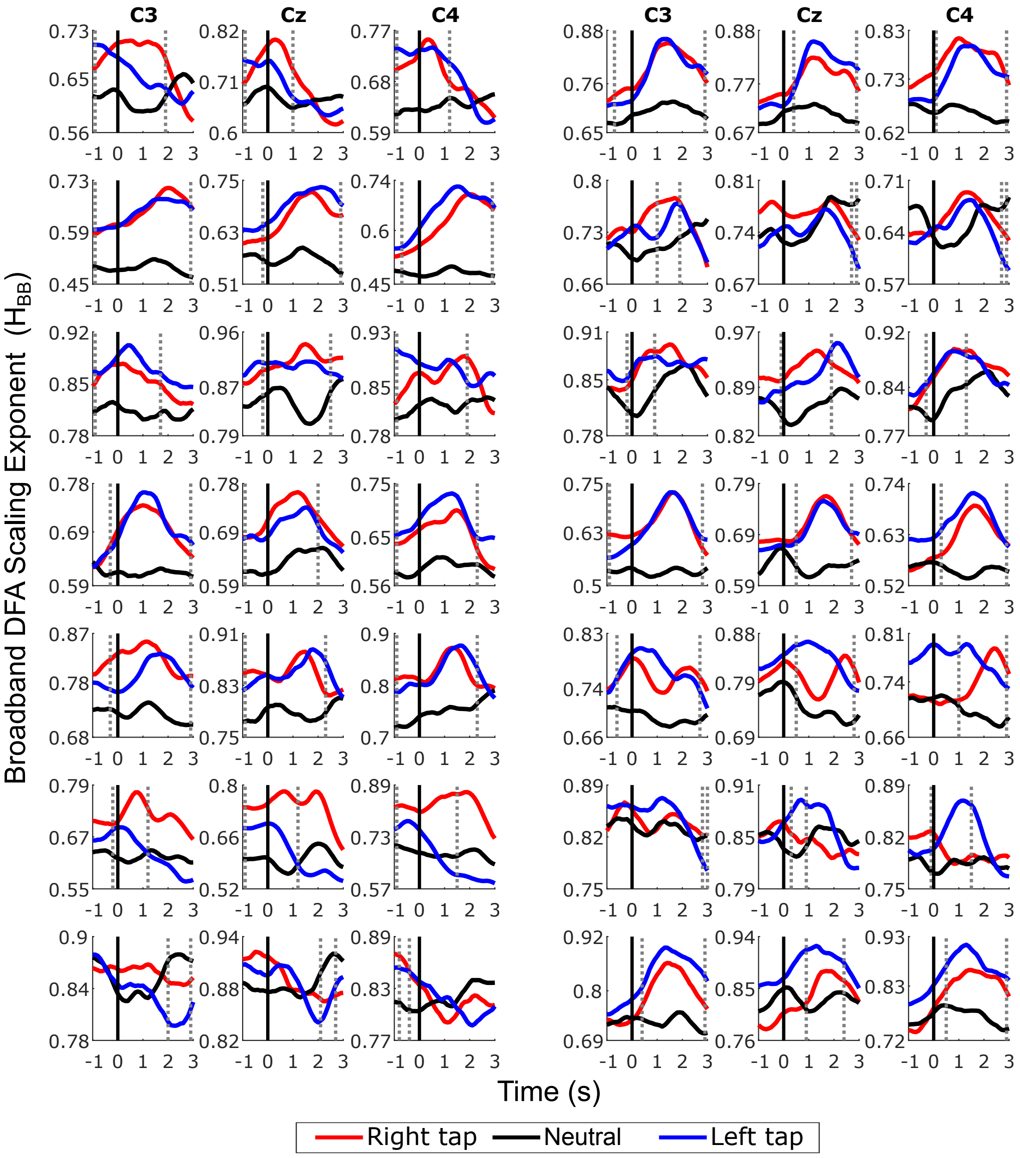


**Figure S1. The time evolution of grand average DFA scaling exponents of broadband EEG ($H_{BB}$) in the individual participants for single finger tap task.** The progressions of mean $H_{BB}$ in individual participants in channels C3, Cz and C4 during right finger tap (red), left finger tap (blue) and resting state (black). The $H_{BB}$ increases during movement intention and execution. The movement onset is at 0 s marked by a solid vertical line. The $H_{BB}$ of movement trials is significantly different from the $H_{BB}$ of resting state trials ($p < 0.05$, Mann Whitney U test)in the time region between the dotted grey vertical lines. A clear increase in $H_{BB}$ is seen during the movement.

## 2 SUPPLEMENTARY FIGURE 2

This figure shows grand average spectrograms of EEG during different types of movements and imagery from finger tapping dataset (Wairagkar et al. (2017)) and EEG Motor Movement/Imagery Dataset (Schalk (2004)). A clear event-related desynchronisation (ERD) (attenuation of power) can be seen prominently in alpha band and to some extent in beta band during all motor tasks after the onset of movement or cue (vertical line). ERD in motor imagery is weaker than motor execution. This helps to validate the datasets.

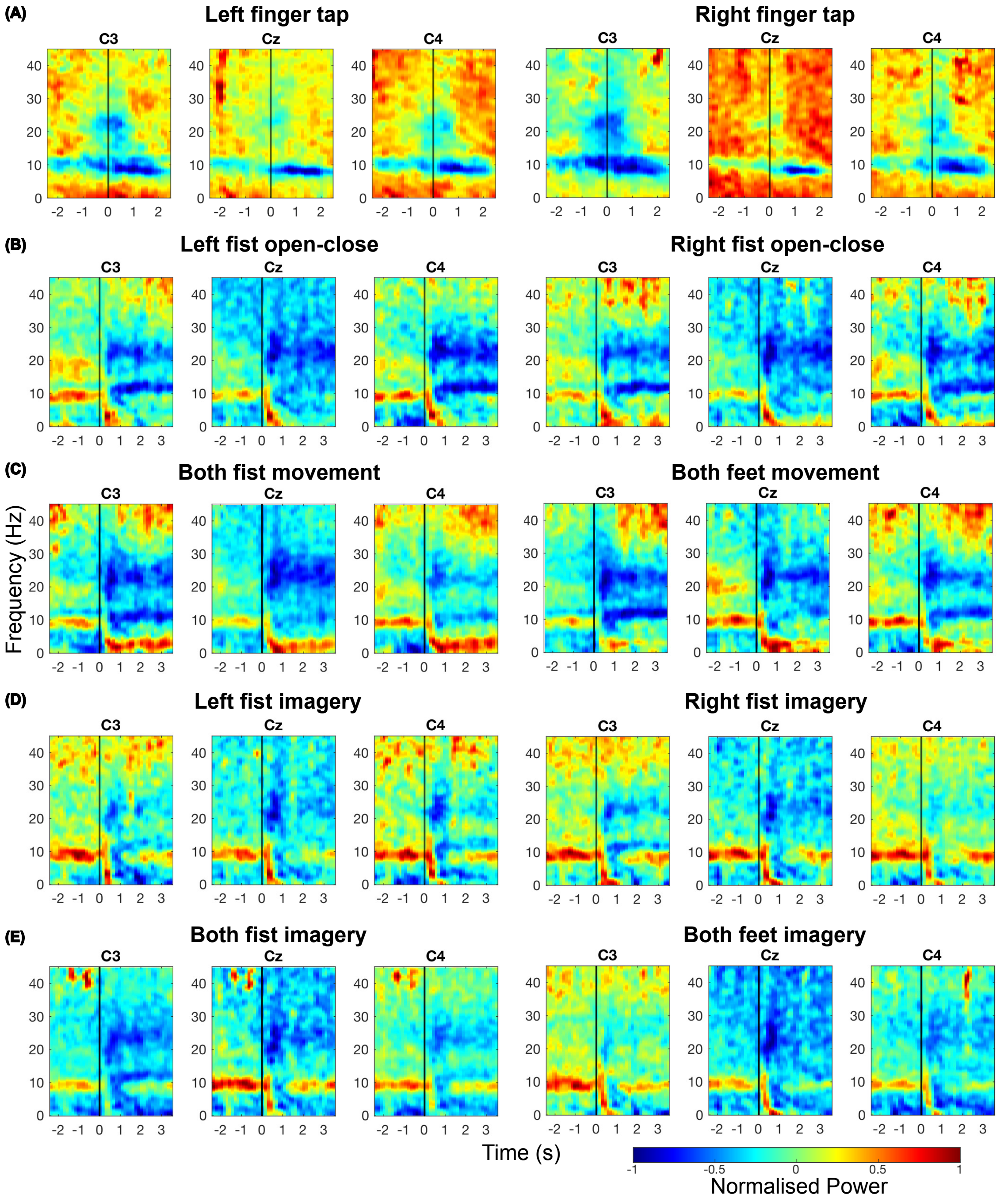


**Figure S2. The grand average spectrograms of EEG during different motor tasks. (A-E)** show the grand average spectrograms in all participants during five different types of motor tasks in channels C3, Cz, and C4. Solid vertical line at 0s marks the onset of finger tap movement in **(A)** and cue for motor execution and imagery in **(B-E)**. A clear ERD is observed in alpha band around 10 Hz in all cases.

## 3 SUPPLEMENTARY FIGURE 3

This figure shows the result of surrogate test by randomly shuffling broadband EEG which confirmed that broadband LRTC ($H_{BB}$) in 2 s EEG windows were destroyed by shuffling. The scaling exponents of the shuffled data are close to 0.5, confirming the presence of white noise with no correlations.

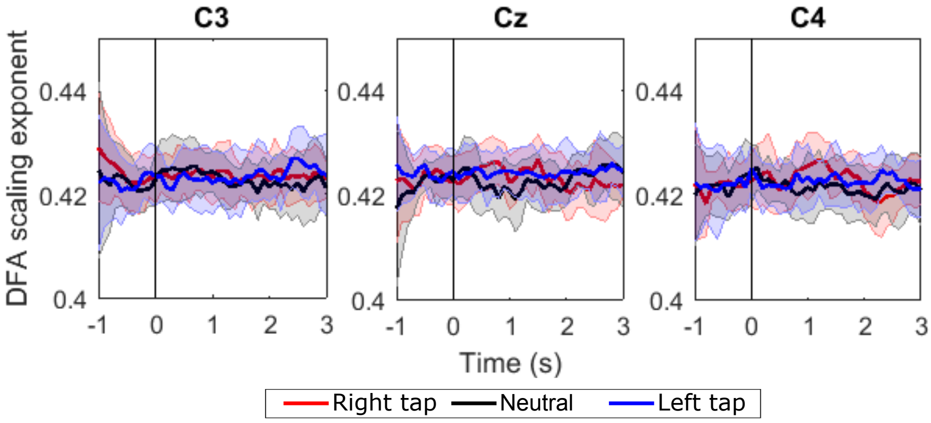


**Figure S3. The time evolution of DFA scaling exponents in the randomly shuffled surrogate data.** The progressions of grand average of mean DFA scaling exponent of the randomly shuffled surrogate windows of all the participants in channels C3, Cz, and C4 during right finger tap (red), left finger tap (blue) and resting state (black). LRTCs are not present in the shuffled data. The movement onset is at 0 s marked by a solid vertical line. The shaded areas show the standard deviation.

## 4 SUPPLEMENTARY FIGURE 4

The broadband LRTC (which increases) and alpha envelope LRTC (which decreases) show changes in opposite directions during motor task and are inversely correlated. These changes in LRTC dynamics occur at the same time as indicated by the strong negative correlation in the scatter plot below of $H_{BB}$ and $H_{alpha}$ using stitched EEG during finger tap and resting state. $H_{BB}$ and $H_{alpha}$ are uncorrelated in resting state.

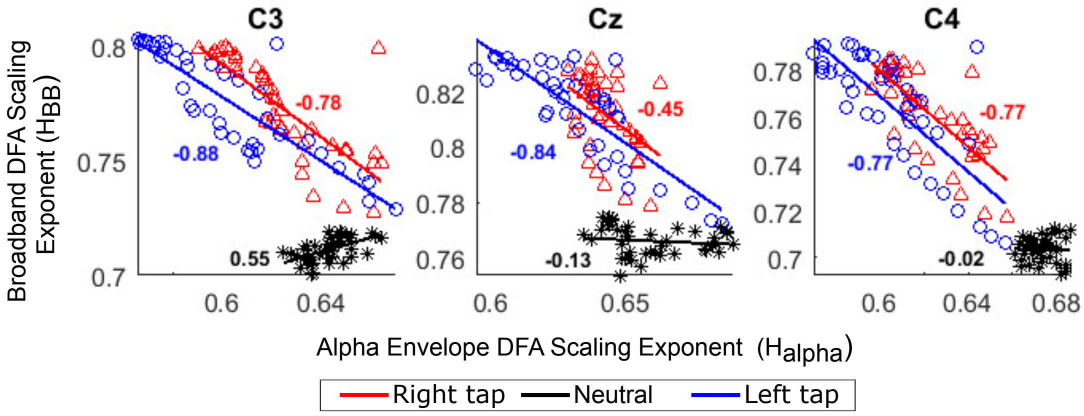


**Figure S4. Correlations between broadband LRTC ($H_{BB}$) and alpha envelop LRTC ($H_{alpha}$) during movement and resting state.** The scatter plot of the grand average $H_{BB}$ and $H_{alpha}$ in channels C3, Cz and C4 is shown with their corresponding correlation coefficients. The correlation coefficients suggest an inverse correlation between $H_{BB}$ and $H_{alpha}$ during right tap (red) and left tap (blue), while there is no correlation during resting state (black).
